# Synthesis, Antiinflammatory and Antibacterial Activity of Novel Indolyl-isoxazoles

**DOI:** 10.4103/0250-474X.59554

**Published:** 2009

**Authors:** S. S. Panda, P. V. R Chowdary, B. S. Jayashree

**Affiliations:** Department of Pharmaceutical Chemistry, Manipal College of Pharmaceutical Sciences, MAHE Manipal-576 104, India

**Keywords:** Antibacterial activity, antiinflammatory activity, indolyl-isoxazoles

## Abstract

Chalcones were synthesized by reacting indole-3-aldehyde, prepared by Vilsemeir Haack reaction with 4-substituted acetophenone in ethanolic KOH solution. These chalcones were immediately reacted with hydroxylamine hydrochloride in presence of glacial acetic acid as reagent to obtain the corresponding isoxazole derivatives. The synthesized heterocycles were characterized on the basis of physical, chemical tests and spectroscopic data. These compounds were tested for the acute antiinflammatory activity and antibacterial activity using carrageenan-induced rat paw edema method and cup-plate method, respectively.

Isoxazoles were reported for their various biological activities[1–3]. The reactive intermediate chalcones involved in their synthesis also exhibit wide range of biological activities[4–6]. The ability of indole to exhibit antiinflammatory, antimicrobial, antifungal activities[7–9] prompted the selection of indole as starting compound. In the light of these interesting biological activities, it appeared of interest to synthesize some new indolyl-isoxazole derivatives and to evaluate their antibacterial and antiinflammatory activities. Indole-3-aldehyde (2) prepared using Vilsemeir Haack reaction by reacting indole (1) with substituted acetophenones (a-j) in ethanolic KOH to obtain chalcones (3a-j), which were condensed with hydroxylamine hydrochloride in presence of sodium acetate and glacial acetic acid to obtain isoxazoles (4a-j). The synthetic sequence leading to the formation of targeted compounds is depicted in Scheme 1.

**Scheme 1:**
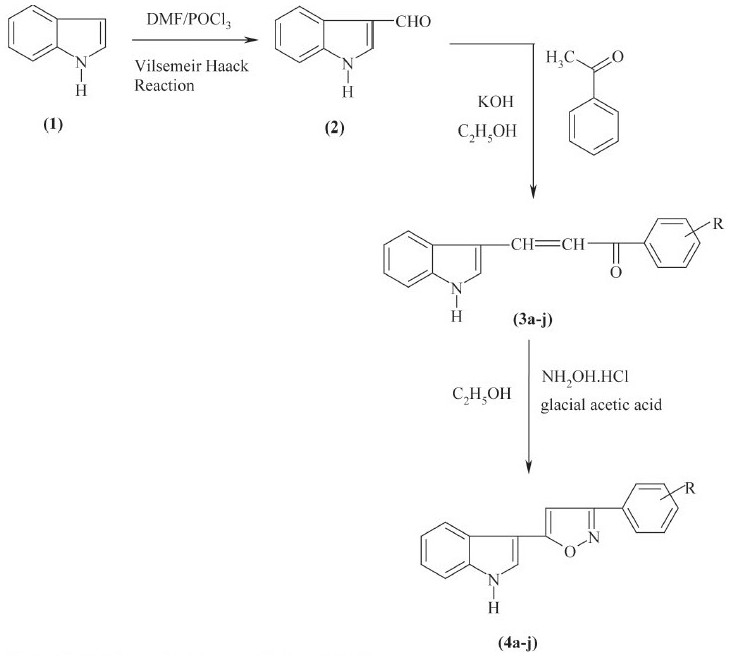
Synthetic scheme for the formation of title compounds

Melting points of the compounds were determined on a Toshniwal electric melting point apparatus and the values were uncorrected. IR spectra of the synthesized compounds were recorded on a Shimadzu-FTIR 8300 using KBr disc method. ^1^H NMR spectra were recorded on a Jeol-GSX 400, (IIT Chennai) using DMSO-d_6_ as solvent. Mass spectra were recorded on a Shimadzu-GCMS 50508. All the solvents used were of analytical grade.

Indole-3-aldehyde (2) and chalcones (3a-j) were prepared following the literature method[10]. The general procedure used for synthesis of 5-(indol-3-yl)-3-(substituted phenyl) isoxazole (4a-j) is as follows. A mixture of chalcone (0.01 mol) and hydroxylamine hydrochloride (0.01 mol) along with sodium acetate and glacial acetic acid in 50 ml ethanol was stirred and refluxed for 8-10 h. The progress of the reaction was monitored by TLC. The reaction mixture was cooled and poured into ice cold water, filtered and dried to get final product, which recrystallized from aqueous ethanol. The solvent system used for TLC was a 8:2 mixture of chloroform:methanol. The yield and melting point are given in Table 1. 5-(Indol-3-yl)-3-(4-methoxyphenyl) isoxazole (4h) IR (KBr) cm^−1^: 3225 (-NH-), 3069 (Ar C-H), 1569 (-C=N-), 1016, 754. ^1^HNMR (DMSO-d_6_): δ 3.82 (s, 3H, -OCH_3_), 6.00 [SUPPORTING:1] (s, 1H, isoxazole ring proton), 7.12 (s, 1H, indolyl proton), 7.41-7.68 (m, 8H, aromatic protons), 11.06 (s, 1H, N-H). Mass m/z: 290 (M^+^), 291 (M^+^ + 1).

**TABLE 1:** PHYSICAL DATA OF 5-(INDOL-3-YL)-3-(SUBSTITUTED PHENYL) ISOXAZOLE

Compd.	R	Mol. formula	M.P (°C)	Rf* Value	Yield (%)
4a	-H	C17H12N2O	147	0.59	77
4b	-NH2 (p)	C17H13N3O	151	0.53	65
4c	-Br (p)	C17H11N2OBr	119	0.45	67
4d	-Cl (p)	C17H11N2OCl	128	0.47	60
4e	-OH (o,p)	C17H12N2O3	175	0.38	75
4f	-F (p)	C17H11N2OF	157	0.41	71
4g	-CH3 (p)	C18H14N2O	138	0.45	58
4h	-OCH3 (p)	C18H14N2O2	183	0.35	78
4i	-OH (p)	C17H12N2O2	161	0.52	72
4j	-NO2 (p)	C17H11N3O3	167	0.57	91

* All the synthesized compounds were recrystallized from ethanol and solvent system in TLC was chloroform:methanol (8:2)

Antiinflammatory activity was measured using the carrageenan-induced paw edema test in rats[11]. Animals were divided into different groups each consisting of six animals. Out of twelve synthesized compounds, five compounds (4a, 4c, 4f, 4g, 4j) were selected as test compounds and standard ibuprofen were administered orally at a dose of 200 mg/kg as an aqueous suspension in 1% CMC, while the control group was fed with the same volume of 1% CMC suspension. The paw volume were measured using a plethysmograph immediately before and 3 h after the carrageenan injection. The percent inhibition of paw volume was calculated by using the formula, percent inhibition= (1-Vt/Vc)×100, where Vt is the mean volume of the test drug, Vc is the mean volume of the control Table 2. The one-way ANOVA (Scheffe's method[12]) test was applied, and the test compounds were found to be significantly active compared to the control (*P* <0.05). The institutional animal ethics committee of Kasthurba Medical College has approved the experimental protocol (No. IACE/KMC/030/2004-05).

**TABLE 2:** ANTIINFLAMMATORY ACTIVITY OF SYNTHESIZED COMPOUNDS

Group	Dose in μg	Mean edema ± SE	% Reduction in edema volume
Control	--	0.406 ± 0.047	--
Ibuprofen	200	0.170 ± 0.019a	68.08
4a	200	0.153 ± 0.021ab	69.5
4c	200	0.268 ± 0.022a	32.9
4f	200	0.105 ± 0.019ab	73.7
4g	200	0.126 ± 0.014ab	70.7
4j	200	0.175 ± 0.026a	36.6

5% allowance value is 0.28 (Scheffe's method), *P<0.05 Vs control. Note: Any two means showing a difference of 0.28 are statistically significant.

All compounds synthesized (4a-4j) were screened for antibacterial activity using the cup-plate agar diffusion method[13] by measuring the zone of inhibition in mm. Ciprofloxacin (50 mg/ml and 100 mg/ml) was used as standard for antibacterial activity. The compounds were screened for antibacterial activity against *S. aureus*, *B. subtilis*, *E. coli* and *P. aeruginosa* in Muller-Hinton agar medium. This sterilized agar medium was poured into Petri dishes and allowed to solidify. On the surface of the media microbial suspensions were spread with the help of sterilized triangular loop. A stainless steel cylinder of 8 mm diameter (pre-sterilized) was used to bore the cavities. All the synthesized compounds (50 mg/ml and 100 mg/ml) were placed serially in the cavities with the help of micropipette and allowed to diffuse for 1 h. Dimethylsulfoxide (DMSO) was used as a solvent for all compounds and as control. The plates were incubated at 37° for 14 h. The zone of inhibition observed around the cups after incubation was measured. The results are presented in Table 3.

**TABLE 3:** ANTIBACTERIAL ACTIVITIES OF SYNTHESIZED COMPOUNDS

Compd.	*S. aureus*		*B. subtilis*		*E. coli*		*P. aeruginisa*	
	50*	100*	50*	100*	50*	100*	50*	100*
4a	12	16	-	-	14	16	11	13
4b	22	26	-	09	14	17	17	24
4c	14	20	-	-	12	14	14	19
4d	13	19	-	-	-	10	13	18
4e	-	14	-	-	-	-	10	14
4f	14	18	-	-	14	16	14	17
4g	-	10	-	-	10	15	12	13
4h	14	21	-	-	12	14	13	17
4i	12	16	-	10	-	-	10	11
4j	20	22	13	15	13	16	16	23
Cipro floxacin	28	34	14	20	15	22	18	25

* Indicates concentration of drug in μg/ml. Zone of inhibition in mm

All six compounds evaluated for antiinflammatory activity exhibited good activity ranging from 36.6 to 73.7% reduction in edema volume. The compounds 4a, 4f and 4g showed significant antiinflammatory activity comparable to ibuprofen and the other compounds 4c and 4j showed moderate activity. The test compounds were found to be significantly active when compared to control group.

The compound 4b and 4j showed good activity against *S. aureus*. Compounds 4b, 4c, 4d and 4j showed moderate activity against *P. aeruginosa*. Compounds 4b, 4c, 4d and 4j did not possess any encouraging activity against *B. subtilis*.
